# Retrograded Resistant Starch Improves Emulsion Stability and Emulsion Gel Properties Stabilized by Myofibrillar Proteins Without Degrading In Vitro Protein Digestibility

**DOI:** 10.3390/foods13233739

**Published:** 2024-11-22

**Authors:** Jinyu Chen, Fangyang Hu, Jiaqi Guo, Wen Zhang, Zijian Wu

**Affiliations:** 1 College of Biotechnology and Food Science, Tianjin University of Commerce, Tianjin 300134, China; 2 Tianjin Key Laboratory of Food Biotechnology, Tianjin 300134, China; 3 Agriculture and Food Engineering College, Baise University, Baise 533000, China

**Keywords:** retrograded resistant starch, myofibrillar proteins, emulsion gel, gel properties, in vitro protein digestibility

## Abstract

The objective of this study was to investigate the effects of retrograded resistant starch (RS3) (0, 2%, 4% and 6%; *w*/*v*) on the emulsion gel properties stabilized by myofibrillar proteins (MPs) and in vitro protein digestibility of the gels. The RS3 was prepared from corn or potato starch using the gelatinization–ultrasound–retrogradation method. The results showed that the addition of RS3 decreased the surface hydrophobicity (*p* < 0.05) and increased the fluorescence intensity of MPs, indicating enhanced protein–protein interactions. More stable emulsions stabilized by MP/RS3 mixtures were formed, along with higher electronegativity, a smaller droplet size and reduced creaming index. These changes promoted the formation of better gel networks with the oil droplets evenly dispersed, thus improving gel strength, water holding capacity (WHC) and texture, especially at the concentration of 6% RS3 added. The gel force results indicated that the addition of RS3 enhanced the hydrophobic interaction and disulfide bonds between MPs. LF-NMR and MRI data further confirmed that RS3 addition facilitated the migration of free water to immobilized water. Furthermore, the incorporation of RS3 caused a relatively lower pepsin digestibility but did not change the overall in vitro protein digestibility of the gels. This paper provides a method to produce high-quality low-GI meat products without degrading protein digestibility.

## 1. Introduction

Most processed meat products (such as emulsified sausages) are actually emulsion gel systems in which fats are dispersed into small balls and stabilized primarily by muscle proteins [1]. To be specific, the fat particles are distributed in the continuous phase formed by meat proteins, and other proteins are absorbed on the surface of the droplets to maintain the stability of the emulsion. Upon heating, disulfide bonds, hydrophobic interaction, hydrogen bonding and other intermolecular forces are formed between the proteins in the continuous phase and between the proteins in the continuous phase and the interfacial proteins, thus resulting in an ordered protein gel network with favorable elasticity and hardness, improved water holding capacity and uniform distribution of the oil droplets. The emulsifying and heat-induced gel properties of myofibrillar proteins (MPs), the major components of muscle proteins, are directly related to the texture and juiciness of manufactured meat [2], and affect the sensory properties of the final products.

In recent years, with the increasing pursuit of low calorie and healthy diets, resistant starch as a new dietary fiber has attracted more and more attention. In addition to the properties of native starch, resistant starch has multiple physiological effects such as maintaining blood sugar stability, promoting lipid metabolism, improving the intestinal environment and promoting mineral absorption [3]. Therefore, it has become an inevitable trend to develop new, healthy and nutritious foods formulated with resistant starch. Furthermore, the distinguished functional properties of resistant starch including hydroscopicity and gelation ability make it applicable in meat processing [4,5]. In general, the physicochemical characteristics of resistant starch are closely related to its natural source and preparation method [6,7]. Nevertheless, there is a lack of studies concerning the influence of different sources of resistant starch on the gel properties of MPs, especially in the emulsion gel system. Moreover, considering that most meat products are processed by heating, the effect of resistant starch on the protein digestibility of heat-induced meat gel appears to be particularly important, since the digestibility of meat protein directly affects its amino acid availability and nutritional value. Recent studies have indicated that protein digestibility is influenced by protein–starch interactions [8,9]. However, the in vitro protein digestion behavior of the emulsion gel stabilized by an MP/resistant starch mixture has rarely been reported.

Generally, resistant starches (RSs) can be divided into five categories: RS1, derived from grains or seeds, is surrounded by a protein/cytoplasm barrier that reduces the availability of enzymes; RS2, mainly present in green bananas, raw potatoes and amylomaize, possesses a compact crystal conformation which prevents the entry and access of enzymes, thus resulting in higher anti-digestibility properties; RS3, known as the retrograded starch, is found in cooked and cooled staple food such as rice, bread and noodles; RS4 refers to the chemically modified starch, such as thermally modified starch, cross-linked starch and phosphorylated starch; and RS5, a complex formed by amylose and lipid, is tough to combine with amylase mainly due to its insolubility and thermal stability [10,11]. Recently, RS3 has attracted more and more attention mainly due to its high safety and easy availability. RS3 is formed by gelatinized starch after cooling, which maintains the basic functional groups of native starch, and it has become the major production type in the resistant starch industry. In addition, it has the advantages of improving texture, appearance and sensory characteristics [12]. In our previous study [13], corn RS3 and potato RS3 were prepared by the gelatinization–ultrasound–retrogradation method for the first time and characterized for their physicochemical properties including morphology, particle size and thermodynamic properties. It was also observed that sausages added with RS3 had a lower estimated glycemic index (eGI). However, the effect of RS3 on the functional properties and in vitro protein digestibility of meat protein-based emulsion gel was unclear.

In the present study, MP from chicken breast muscle was treated with corn RS3 and potato RS3, respectively, at different concentrations (0, 2%, 4% and 6%; *w*/*v*). The surface hydrophobicity, intrinsic fluorescence characteristics and average particle size of the MP/RS3 mixtures were determined, and the impact of RS3 on emulsion stability and emulsion gel properties stabilized by MPs were systematically investigated by measuring the zeta potential, creaming index, apparent viscosity, dynamic rheology, gel strength and texture, water holding capacity, water distribution, intermolecular forces and microstructure. The in vitro protein digestion behavior of the composite emulsion gels was also discussed. Our findings provide a theoretical basis for developing high-quality functional meat products with low GI.

## 2. Materials and Methods

### 2.1. Materials

Fresh chicken breast muscle was obtained from a supermarket (China Resources Vanguard Supermarket, Tianjin, China) and kept at 4 °C before use. Corn and potato starches were provided by Liangshan Linghua Biotechnology Co., Ltd. (Jining, China) and Beijing Gusong Trade Co., Ltd. (Beijing, China), respectively. Bromophenol blue (BPB) was purchased from Tianjin Kemi Ou chemical reagent development center (Tianjin, China); simulated saliva (pH 7.0), gastric fluid (pH 3.0) and intestinal fluid (pH 7.0) were purchased from Sigma-Aldrich (St. Louis, MO, USA); and potassium bromide (spectral purity), Nile red, β-mercaptoethanol and other analytical reagents were purchased from Maclin Biochemical Technology Co., Ltd. (Shanghai, China).

### 2.2. Preparation and Characterization of RS3

RS3 was prepared from corn or potato starch using the gelatinization–ultrasound–retrogradation method according to our previous method [13]. Starch suspensions (40%, *w*/*v*) were incubated in a 95 °C water bath for 30 min. After cooling, the gelatinized starches were treated with ultrasound (70 W) using a CE-9600 portable ultrasonic instrument (Jiekang Ultrasonic Equipment Co., Ltd., Dongguan, China) at 40 °C for 30 min and then kept at 4 °C for 24 h for retrogradation. Subsequently, the starches were allowed to stand for 1 h at room temperature, dried to constant weight at 105 °C, crushed and sifted (100 mesh) to obtain RS3.

The average size of the RS3 particles was determined using a Mastersizer 3000E analyzer (Malvern, UK) at room temperature. The samples were dispersed in distilled water to obtain a concentration of 1% (*w*/*v*). The test conditions were as follows: the refractive index of particles and distilled water (the dispersion system) was 1.50 and 1.330, respectively, and the absorption rate of particles was 0.01.

The amylose content of the RS3 samples was determined according to a previous study [14]. Briefly, the RS3 sample (10 mg) was dispersed in 9 mL of NaOH aqueous solution (1 mol/L) and 1 mL of anhydrous ethanol. The mixture was incubated at 100 °C for 10 min, cooled to room temperature, and then diluted with distilled water to a total volume of 100 mL. The diluted solution (5 mL) was mixed with 1 mL of acetic acid solution (1 mol/L) and 1 mL of iodine solution (0.2%), and kept at room temperature for 20 min. The absorbance was determined at 620 nm using a spectrophotometer (U-5100, Hitachi, Chiyoda, Japan). The amylose content was calculated from a standard curve established with potato amylose (Sigma-Aldrich).

### 2.3. Extraction of MP

MP was extracted from chicken breast muscle based on the procedures previously reported [15]. The fresh meat (200 g) was minced, mixed with 800 mL of extracting solution, which contained 0.1 mol/L NaCl, 2 mmol/L MgCl_2_, 1 mmol/L EDTA-2Na and 0.1 mol/L phosphate buffer (pH 7.0), and then homogenized at 11,000 rpm using an agitator (POLYTRON, Kinematica, Malters, Switzerland). The homogenate was centrifuged at 4500× *g* for 15 min at 4 °C using an Avanti J-E centrifuge (Beckman Coulter, Krefeld, Germany). The deposit was treated with the extracting solution two more times. Ultimately, the obtained protein sediment was washed three times with 800 mL of NaCl (0.1 mol/L) using the above centrifugal condition. The concentration of protein was measured to be 10% (*w*/*w*) using the Biuret method.

### 2.4. Preparation of MP/RS3 Mixtures

According to the literature [16], the final protein concentration for MP/polysaccharide composite emulsions and emulsion gels was 20 mg/mL. Therefore, this concentration was selected to prepare MP/RS3 mixtures in order to facilitate the preparation of MP/RS3 composite emulsions and emulsion gels. Briefly, MP/RS3 mixtures (20 mg/mL, final protein concentration) were prepared by dispersing the MP pellet into RS3 suspensions (0%, 2%, 4% and 6%, *w*/*v*), using 0.6 mol/L NaCl as solvent. The MP/RS3 mixtures were stirred thoroughly at 4 °C for 12 h for further analysis.

### 2.5. Surface Hydrophobicity of MP/RS3 Mixtures

Briefly, MP/RS3 mixtures were diluted to 2 mg/mL (protein concentration) with 20 mmol/L phosphate buffer (pH 6.0). Then, 2 mL of the diluted samples was mixed with 400 μL of 1 mg/mL bromophenol blue [17]. After reaction for 5 min, the mixtures were centrifuged at 3000× *g* for 20 min using an Avanti J-E centrifuge (Beckman Coulter, Krefeld, Germany). The supernatants were diluted 50 times and the absorbance was determined at 595 nm using a spectrophotometer (U-5100, Hitachi, Chiyoda, Japan). Phosphate buffer (20 mmol/L, pH 6.0) without protein was regarded as a control. The surface hydrophobicity was expressed as the amount of BPB bound to surface hydrophobic groups of the protein and calculated according to Equation (1):

(1) $$\mathrm{BPB}\;\mathrm{bound}\;(\mu g)=\frac{A_{\mathrm{control}}-A_{\mathrm{sample}}}{A_{\mathrm{control}}}\times 400\;\mu g$$

where *A*_control_ and *A*_sample_ represent the absorbance values at 595 nm of the control and samples, respectively.

### 2.6. Endogenous Tryptophan Fluorescence of MP/RS3 Mixtures

The endogenous tryptophan fluorescence of MP/RS3 mixtures (0.5 mg/mL, final protein concentration, diluted with 0.6 mol/L NaCl) was determined using an F-4600 fluorescence spectrophotometer (Hitachi, Tokyo, Japan). Emission spectra in the wavelength range of 300–400 nm were recorded, with the excitation wavelength at 283 nm and a scanning rate of 1200 nm/min.

### 2.7. Particle Size Distribution of MP/RS3 Mixtures

The particle size of MP/RS3 mixtures was determined using a Mastersizer 3000E analyzer (Malvern, UK) at room temperature [15]. The samples were diluted to 5 mg/mL (protein concentration) with 0.6 mol/L NaCl, and dispersed in distilled water with the refractive index of 1.330.

### 2.8. Preparation of Emulsions Stabilized by MP/RS3 Mixtures

Soybean oil was added to MP/RS3 mixtures (20 mg/mL) in a volume ratio of 1:4 and the mixture was homogenized for 1 min at 8000 rpm with a homogenizer (FLUKO FA 25, Shanghai, China) to obtain oil-in-water (O/W) emulsions [17].

### 2.9. Determination of Emulsion Properties

#### 2.9.1. Zeta Potential

The zeta potential of emulsions stabilized by MP/RS3 mixtures was measured using a Zetasizer Nano-ZS instrument (Malvern, UK) at room temperature. The emulsion samples were diluted to 0.5 mg/mL (protein concentration) with 0.6 mol/L NaCl, and then transferred into disposable capillary cells (Malvern, UK) for determination.

#### 2.9.2. Creaming Index

The emulsion samples were transferred into calibrated glass tubes and kept at room temperature for 24 h [2]. The creaming index was expressed as the percentage of the height of the subnatant to the initial total height of the emulsion.

### 2.10. Preparation of MP/RS3 Composite Emulsion Gels

The fresh emulsions (15 mL) prepared in Section 2.8 were placed into a glass vial (inner diameter 25 mm, height 50 mm), incubated from 20 to 75 °C at a heating rate of 1 °C/min and kept at 75 °C for 10 min. The gels were then cooled down and maintained at 4 °C overnight [17]. The gels were equilibrated at room temperature for 30 min before function analysis.

### 2.11. Determination of Emulsion Gel Properties

#### 2.11.1. Rheological Properties

The apparent viscosity and dynamic viscoelasticity of the emulsions stabilized by MP/RS3 mixtures were measured using an MCR 301 rheometer (Anton Paar, Graz, Austria) equipped with two parallel plates and a PP 50 conical probe (25 mm diameter) according to a previous study [2]. The emulsion sample (5 g) was evenly spread on the lower plate and the test gap was 1.0 mm. The changes in apparent viscosity were monitored as the shear rate increased from 0 to 120 s^−1^ at 25 °C. For a temperature sweep, the viscoelastic properties of the emulsions were determined in terms of storage modulus (G′) and loss modulus (G′′) by heating the samples from 20 to 80 °C at a rate of 2 °C/min, with a fixed frequency of 0.1 Hz and a maximum strain of 0.01 in the linear viscoelastic region.

#### 2.11.2. Gel Strength and Texture

The gel strength of the MP/RS3 composite emulsion gels was measured at a constant test speed of 1.0 mm/s using a TA.XT Plus texture analyzer (Stable Micro System, Godalming, UK) coupled with a cylinder probe (P/0.5) at 25 °C [15]. In addition, a texture profile analysis (TPA) procedure was employed for the determination of textural parameters including hardness, elasticity, cohesiveness, gumminess, chewiness and resilience. The measurement parameters were as follows: 1.0 mm/s pre-test speed, 0.5 mm/s test speed, 1.0 mm/s post-test speed, 5.0 g trigger force and 50% compression ratio. The emulsion gels were compressed by two cycle tests with a 5 s interval.

#### 2.11.3. Water Holding Capacity (WHC)

A total of 6 g of MP/RS3 composite emulsion gels was placed into a 10 mL centrifuge tube and centrifuged at 10,000× *g* for 10 min using an Avanti J-E centrifuge (Beckman Coulter, Krefeld, Germany). WHC (%) was represented as the weight of the precipitation as a percentage of the weight of the initial gel [17].

#### 2.11.4. Water Distribution in Emulsion Gels

Transverse relaxation time (*T*_2_) was determined by a low field nuclear magnetic resonance (LF-NMR) analyzer (NMI 20-025-V-I, Niumag, Shanghai, China) according to a previous study [15]. Briefly, a total of 2 g of the MP/RS3 composite emulsion gels was placed into a nuclear magnetic tube with a diameter of 25 mm, and then subjected to the instrument for analysis. Carr–Purcell–Meiboom–Gill (CPMG) pulse sequences were used to measure the spin–spin *T*_2_ data, with the time of echoes (TE) of 1 ms and number of echoes (NECH) of 18,000. The time waiting (TW) and number of scans (NS) were 3500 ms and 4, respectively.

The distribution of water in the gels was further observed by magnetic resonance imaging (MRI) (MicroMR-25, Niumag, China). Multiple spin echo sequences with a TE value of 20 ms and a TR value of 500 ms were used to obtain the images of proton density, which were transferred to the pseudo-color pictures using Niumag Image Evaluation software (Version 3.0).

#### 2.11.5. Molecular Forces

The forces involved in the formation of MP/RS3 composite emulsion gels were evaluated as previously reported [2]. The gel sample was chopped and homogenized in denaturing solutions (0.05 mol/L NaCl, to assess nonspecific association; 0.6 mol/L NaCl, to analyze ionic interaction; 0.6 mol/L NaCl + 1.5 mol/L urea, to probe hydrogen bonds; 0.6 mol/L NaCl + 8.0 mol/L urea, to detect hydrophobic interaction; and 0.6 mol/L NaCl + 8.0 mol/L urea + 0.5 mol/L β-mercaptoethanol, to indicate disulfide bonds). The homogenates were kept at room temperature for 1 h, and then subjected to centrifugation at 10,000× *g* for 10 min using an Avanti J-E centrifuge (Beckman Coulter, Germany). The content of protein in the supernatants was determined by the Biuret method. The molecular forces were expressed as the soluble protein content in the denaturing solutions (g/L).

#### 2.11.6. Microstructure

The distribution of oil droplets in the emulsion gels was observed by a FY3000 confocal laser scanning microscope (Olympus, Tokyo, Japan) according to a previous study [18]. Briefly, MP/RS3 composite emulsion gels were cut into thin slices (1 mm thick) with a microtome (CM-1850, Leica, Wetzlar, Germany), and then stained with a mixture of Nile blue (1 mg/mL, for protein) and Nile red (1 mg/mL, for oil droplets) for 5 min. After staining, the samples were observed by the microscope (10× objective) with excitation wavelengths of 633 nm and 488 nm for Nile blue and Nile red, respectively.

### 2.12. In Vitro Protein Digestion of Emulsion Gels

In vitro protein digestion of the composite emulsion gels was carried out as previously described [17,19] with slight modifications. The gel was mixed with simulated saliva fluid (pH 7.0) without amylase at 20 mg of protein per milliliter, homogenized and kept at 37 °C for 2 min. The solution was then added to the same volume of simulated gastric fluid (pH 3.0) containing pepsin from porcine gastric and incubated at 37 °C for 1 h. The gastric digestion was terminated by raising the pH to 7.0 with 1 mol/L NaOH using a pH meter (EL 20, Mettler Toledo, Greifensee, Switzerland). An equal volume of simulated intestinal fluid (pH 7.0) containing pancreatin from porcine pancreas and porcine bile extract was subsequently mixed with the gastric phase and kept at 37 °C for 2 h. All digestive processes were conducted in a shaking incubator (SHA-B, Lichen Technology, Shanghai, China). The reaction was stopped by heating the corresponding digest in a boiling water bath for 5 min. After centrifugation (4 °C, 10,000× *g*, 10 min), the nitrogen (N) content in the supernatant was measured by the Kjeldahl method and protein digestibility (%) was calculated according to Equation (2):

(2) $$\mathrm{Digestibility}(\%)=\frac{W_{1}}{W_{0}}\times 100$$

where *W*_1_ is the N content in the supernatant and *W*_0_ is the N content in the gel sample.

The particle size of the protein digests after simulated saliva/gastric digestion and saliva/gastric/intestinal digestion, respectively, was determined using a Mastersizer 3000E analyzer (Malvern, UK).

### 2.13. Statistical Analysis

All determinations were performed at least three times, and data were expressed as mean ± standard deviation. One-way ANOVA followed by Duncan’s test of SPSS version 22.0 (IBM software, Armonk, NY, USA) was used for statistical analysis, and *p* < 0.05 was considered as significant. Origin 2022 (Origin Lab, Northampton, MA, USA) was used to plot figures and carry out correlation analysis.

## 3. Results and Discussion

**Figure 1:**
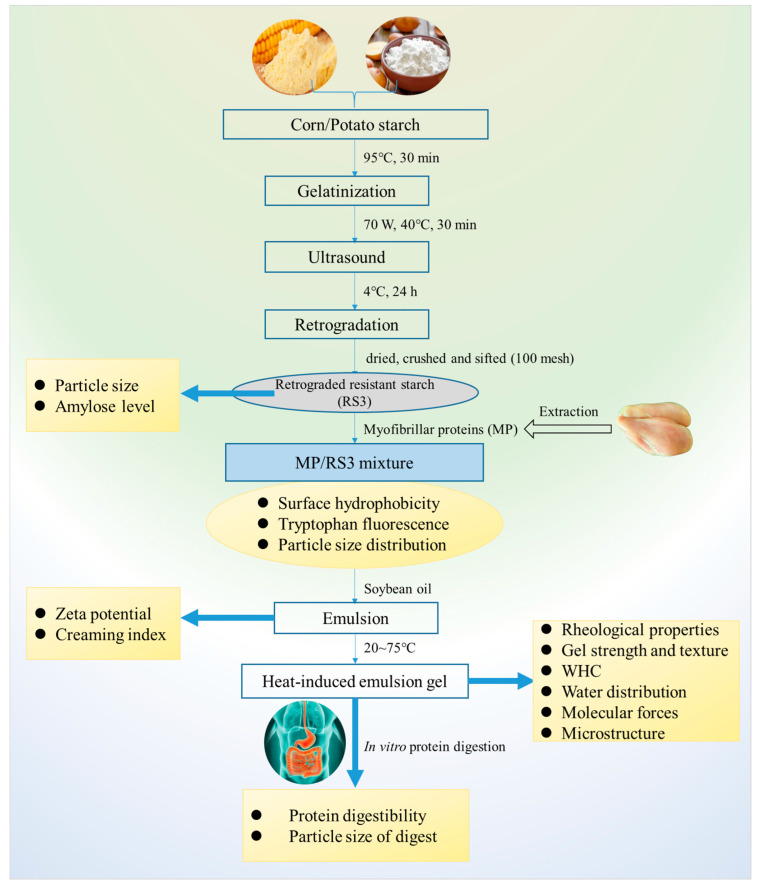
Flow chart of preparation and characterization of emulsion gels stabilized by MP/RS3 mixtures.

Figure 1 shows the flow chart of the preparation and characterization of emulsion gels stabilized by MP/RS3 mixtures. In the following study, the surface hydrophobicity, tryptophan fluorescence and particle size of MP/RS3 mixtures were determined, and the impact of RS3 on emulsion stability and emulsion gel properties stabilized by MPs were investigated. The in vitro protein digestion behavior of the composite emulsion gels was also discussed.

### 3.1. Effect of RS3 Addition on Surface Hydrophobicity of MP

**Figure 2:**
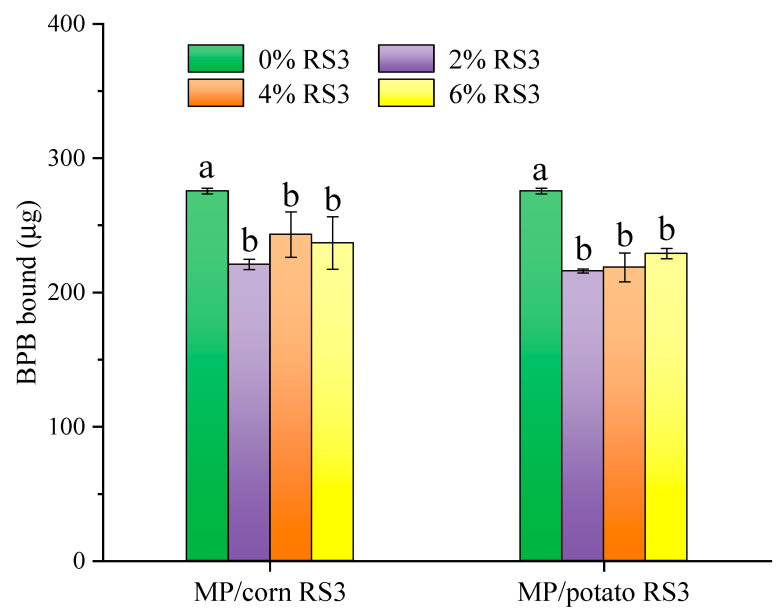
Surface hydrophobicity of MP and MP/RS3 mixtures. Different letters in each group indicate significant difference (*p* < 0.05).

The amount of protein bound BPB is regarded as a suitable parameter for rapid evaluation of protein surface hydrophobicity since hydrophobic BPB can bind to the hydrophobic sites in proteins. As shown in Figure 2, the surface hydrophobicity of MPs added with corn RS3 and potato RS3, respectively, significantly decreased (*p* < 0.05) compared to that of pure MPs (275.54 ± 2.10 μg), and there was no significant difference among the protein samples blended with different amounts of RS3 (2–6%). RS3 containing a great number of hydroxyl groups had potential to form hydrogen bonds with water, which might reduce protein–water interaction and enhance protein–protein interaction. The hydrophobic core formed by nonpolar amino acids became tight, and the exposed hydrophobic groups decreased, thus reducing the surface hydrophobicity of the proteins. Moreover, it has been reported that the increased protein–protein interaction might result in the destruction of some nonpolar amino acids, which could also reduce the protein’s surface hydrophobicity [20].

### 3.2. Effect of RS3 Addition on Tryptophan Fluorescence of MP

**Figure 3:**
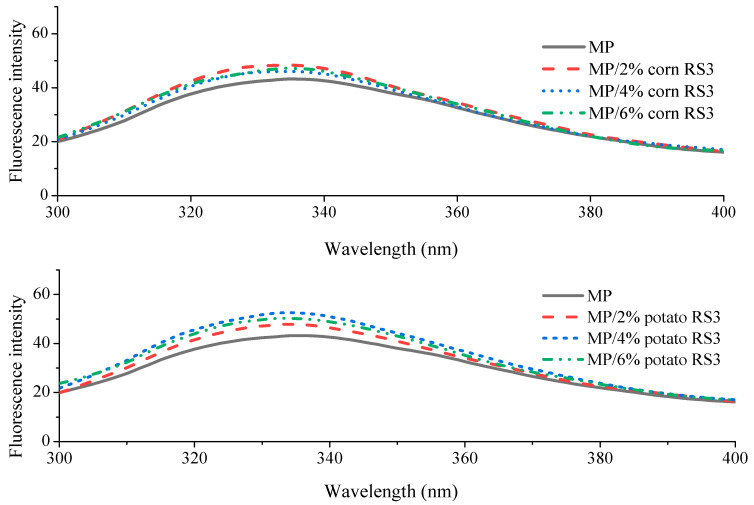
Intrinsic tryptophan fluorescence spectra of MP and MP/RS3 mixtures.

The intrinsic fluorescence characteristics of MPs, including tryptophan fluorescence intensity and maximum emission wavelength (λ_m_), were determined and presented in Figure 3. The addition of RS3 (2–6%) prepared from corn or potato starch increased the fluorescence intensity of MPs, along with slight blue shifts in λ_m_ (337→335 nm for corn RS3 and 337→332 nm for potato RS3). These results indicated that the microenvironment around tryptophan residues was slightly changed. This could be due to enhanced intermolecular cross-linking of the proteins caused by RS3 addition, which was in accordance with the results of surface hydrophobicity (Figure 2). The tryptophan residues were located in a more hydrophobic core, and therefore had a higher fluorescence intensity and lower λ_m_.

### 3.3. Particle Size Distribution of MP/RS3 Mixtures

**Figure 4:**
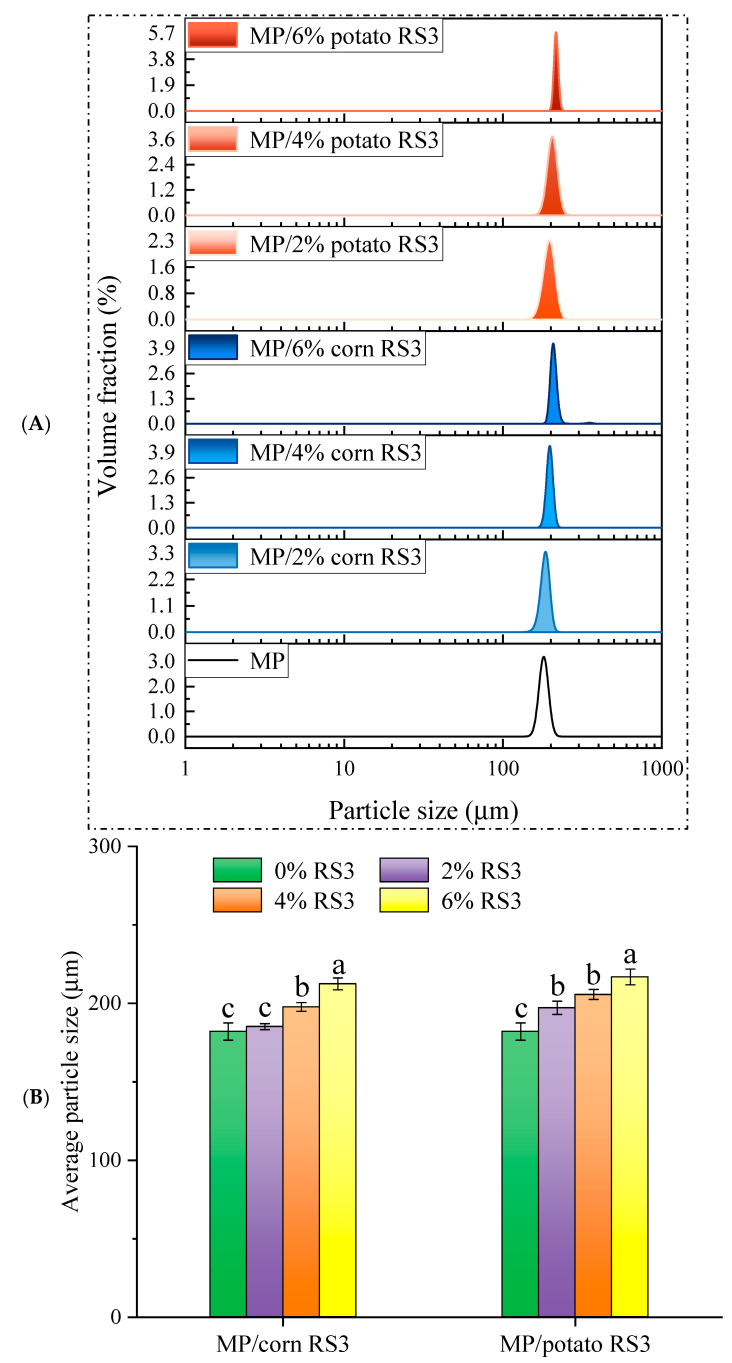
Particle size distribution (**A**) and average particle size (**B**) of MP and MP/RS3 mixtures. Different letters in each group indicate significant difference (*p* < 0.05).

The particle size distribution and average size of the MP/RS3 mixtures are illustrated in Figure 4. The presence of 4% RS3 significantly increased the average particle size of MPs (*p* < 0.05), and the average diameter of the MP/RS3 mixtures was further improved when 6% RS3 was added. As the amount of RS3 added rose, the hydrophobic interaction, hydrogen bonding and other intermolecular forces between MP and RS3 might induce the formation of larger complexes, which led to the increase in particle size. A previous study also reported that the average size of hairtail myosin was increased by the addition of corn starch with different amylose levels [21]. Moreover, in the following study, potato RS3 had a higher average particle size than corn RS3. It was not difficult to explain why the average particle size of MP/potato RS3 was larger than that of MP/corn RS3 in the range of 2–6% RS3 added.

### 3.4. Properties of Emulsions Stabilized by MP/RS3 Mixtures

#### 3.4.1. Zeta Potential

**Figure 5:**
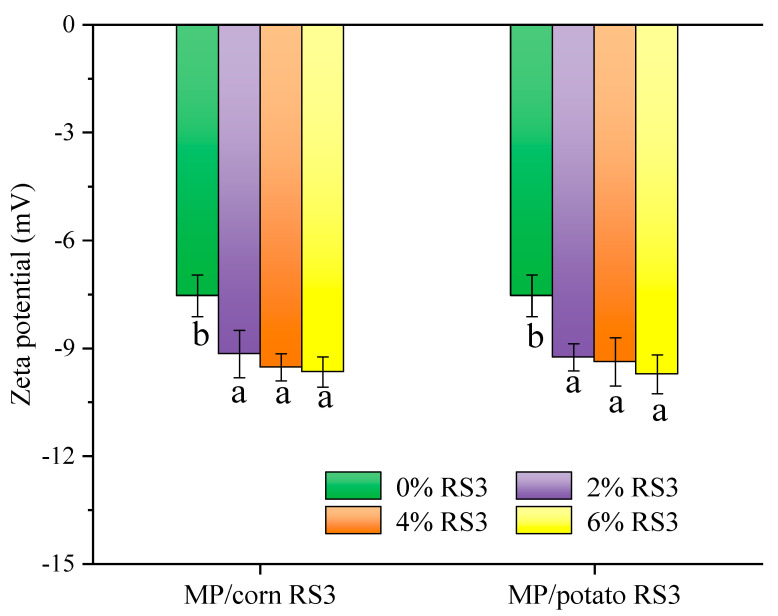
Zeta potential of the emulsions stabilized by MP or MP/RS3 mixtures. Different letters in each group indicate significant difference (*p* < 0.05).

Zeta potential is commonly used to indicate the charge of emulsion droplets and the stability of emulsion. Generally speaking, the greater the absolute value of zeta potential, the more stable the emulsion. Figure 5 shows the zeta potential of the emulsions stabilized by MP/RS3 mixtures. Since the pH of the system (pH 7.0) was far from the isoelectric point of MPs (pH 5.2), the zeta potential of the pure MP emulsion showed as being electronegative, which was close to previous findings [2]. After adding RS3, the zeta potential of the emulsions stabilized by MP/RS3 mixtures decreased with the increase in the amount of RS3 (2–6%). Our preliminary work indicated that the prepared RS3 used in the present study was electronegative. Therefore, it can be inferred that the negatively charged RS3 was adsorbed onto the surface of MP-coated droplets, which enhanced electrostatic repulsion between the droplets and further improved the emulsion stability.

#### 3.4.2. Stability of Emulsions

**Figure 6:**
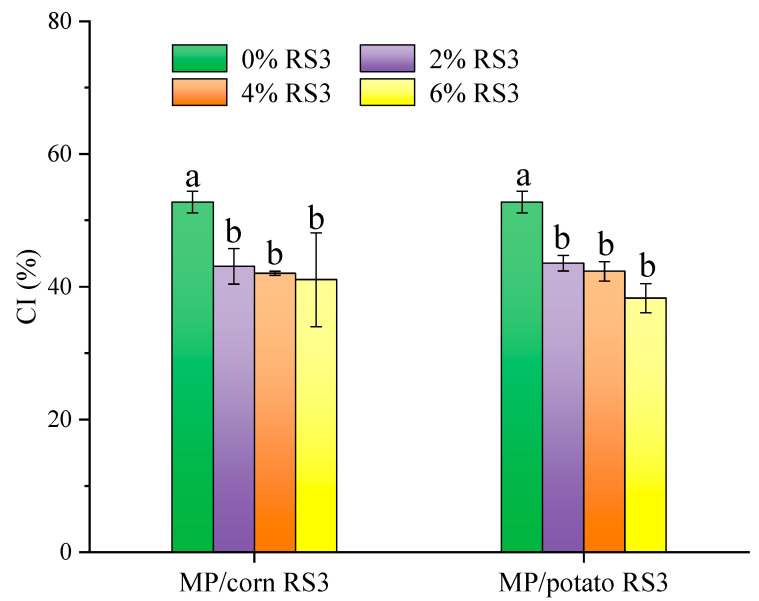
Creaming index of the emulsions stabilized by MP or MP/RS3 mixtures. Different letters in each group indicate significant difference (*p* < 0.05).

Due to the density difference between the continuous phase and dispersed phase of an emulsion, the oil droplets move upward under gravity or centrifugal force and form a cream layer at the top [22]. In general, the higher the creaming index (CI), the worse the emulsion stability. Thus, the stability of the emulsions stabilized by MP/RS3 mixtures during storage can be evaluated by the changes in CI. As presented in Figure 6, the CI of MP/RS3 composite emulsions was significantly (*p* < 0.05) lower than that of pure MP emulsion, with the lowest values of 41.06% (6% corn RS3 added) and 38.28% (6% potato RS3 added), respectively, which were approximately reduced by 25% as compared to that of the pure emulsion. This indicated that RS3 addition improved the stability of MP-based emulsions. The adsorption of RS3 onto the surface of MP coatings might prevent the coalescence of oil droplets and the phase separation of the emulsions by increasing electrostatic repulsion and steric hindrance between the droplets [2,23]. In addition, RS3 may not only act as a stabilizer to increase the thickness of the protein layers that coated oil droplets, but also absorb water and swell to form a stable cross-linked network structure with proteins, so as to improve the stability of the emulsions [24]. This was consistent with the analysis of zeta potential (Figure 5). According to the literature [25], the interactions between protein and starch involved non-covalent forces such as electrostatic interaction, hydrogen bonding, hydrophobic interaction and van der Waals force. In this study, the particle size results (Figure 4) indicated that the large-sized complexes might be formed through the non-covalent interactions between MP and RS3. In addition, RS3 may bind to positively charged groups (such as NH_3_^+^) of amino acid side chains via electrostatic interaction. A similar reduction in CI was observed by adding chitosan to MP emulsions, which stabilized oil droplets and delayed their movement, mainly due to the electrostatic repulsion, steric hindrance and thickening effect caused by chitosan [26].

Generally, a large number of hydrophilic and hydrophobic groups are distributed in MP chains. The hydrophobic groups promote the adsorption of some proteins on the surface of oil droplets to form an interfacial layer, while the proteins in the continuous phase interact with the interfacial proteins through hydrogen bonding and hydrophobic interaction, thus stabilizing the oil droplets. The addition of RS3 may increase the electrostatic repulsion of the emulsion system, promote the diffusion of MPs at the oil–water interface and allow more proteins to be adsorbed on the interfacial layer. The adsorbed proteins contributed to the formation of rigid interfacial films, while those un-adsorbed enhanced the viscosity of the continuous phase due to the RS3 binding, thereby collectively improving the stability of the emulsion [2]. This was further confirmed by the subsequent measurement of apparent viscosity.

### 3.5. Properties of Emulsion Gels Stabilized by MP/RS3 Mixtures

#### 3.5.1. Apparent Viscosity

**Figure 7:**
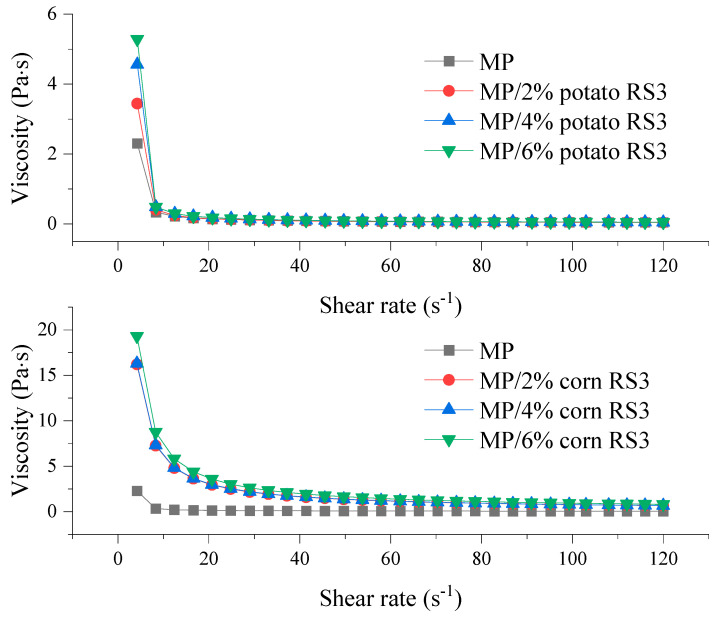
Apparent viscosity of the emulsions stabilized by MP or MP/RS3 mixtures.

The apparent viscosity of the emulsions stabilized by MP/RS3 mixtures was evaluated by the steady shear rheological test and is shown in Figure 7. The apparent viscosity of all emulsions declined with the increase in shear rate and then tended to be gentle, exhibiting an obvious shear-thinning behavior. This could be explained by the destruction of the protein network structure [26] and flocculation of oil droplets [17]. The addition of RS3 increased the apparent viscosity of the MP emulsion, in a concentration-dependent manner. It is generally accepted that the viscosity of the emulsion largely depends on the particle size and conformation of protein molecules and their interactions [26]. Based on the results of surface hydrophobicity (Figure 2) and average particle size (Figure 4), the presence of RS3 might facilitate the formation of MP cross-linking, which increased the molecular size and entanglement among the proteins. The enhanced viscosity of the emulsion system in turn prevented the migration and flocculation/coalescence of the fat droplets. Thus, the incorporated oil could function as a filler, leading to the formation of a firm gel structure [17].

#### 3.5.2. Temperature Ramp Test

**Figure 8:**
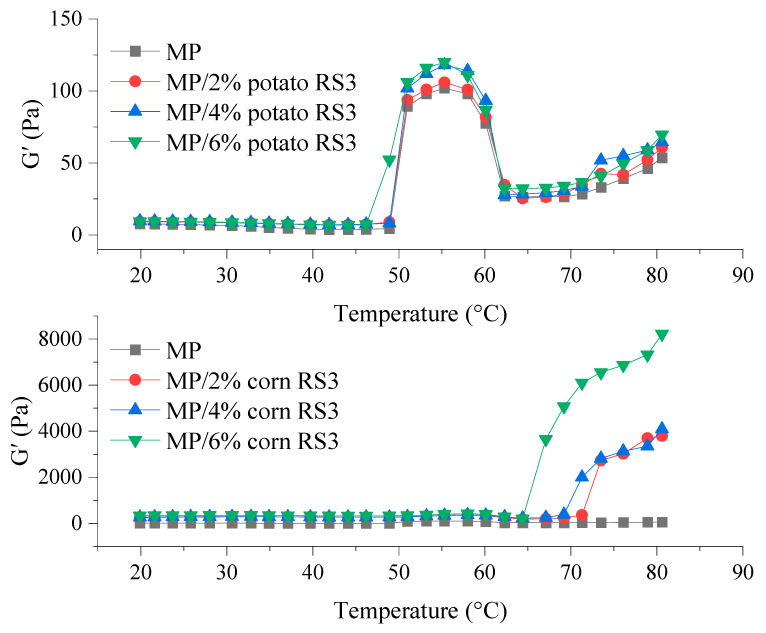
Storage modulus (G′) of the emulsions stabilized by MPs or MP/RS3 mixtures.

The viscoelasticity and gelling performance of the emulsions stabilized by the MP/RS3 mixtures during the thermal process (20–80 °C) were evaluated by dynamically monitoring the changes in storage modulus (G′) (Figure 8). During heating, MP emulsions underwent three stages in terms of gel setting, gel weakening and gel reinforcing, involving disulfide bonds, hydrophobic interaction, hydrogen bonding and other associations [2]. In the first stage (around 50–55 °C), the G’ of all samples increased, indicating the initial formation of gel structure, mainly due to the cross-linking of denatured myosin heads. With the temperature rising to 65 °C, the denaturation and aggregation of myosin tails might break down the established protein network, leading to a decrease in G′. The subsequent increase was observed in the temperature range of 65–80 °C, indicating that a firm and irreversible three-dimensional gel network was formed.

As shown in Figure 8, the G′ in magnitude increased with the increment in the RS3 added (2–6%). In particular, the final G’ value of the emulsion treated with 6% corn RS3 reached 8220 Pa, approximately 153 times that of the pure emulsion, indicating that the elasticity and rigidity of the protein gel composited with corn RS3 were markedly improved. According to the results of surface hydrophobicity (Figure 2), the addition of RS3 may favor the development of an entanglement network via protein–protein interactions, facilitating the fat particles being better anchored into the protein matrix. On the other hand, RS3 could fill in the interstitial spaces of the MP network, and bind water and fat to form a strong gel structure with high elasticity. Psyllium husk dietary fiber was also reported to enhance gel elasticity by promoting the interactions between MPs [27].

#### 3.5.3. Gel Strength and WHC

**Figure 9:**
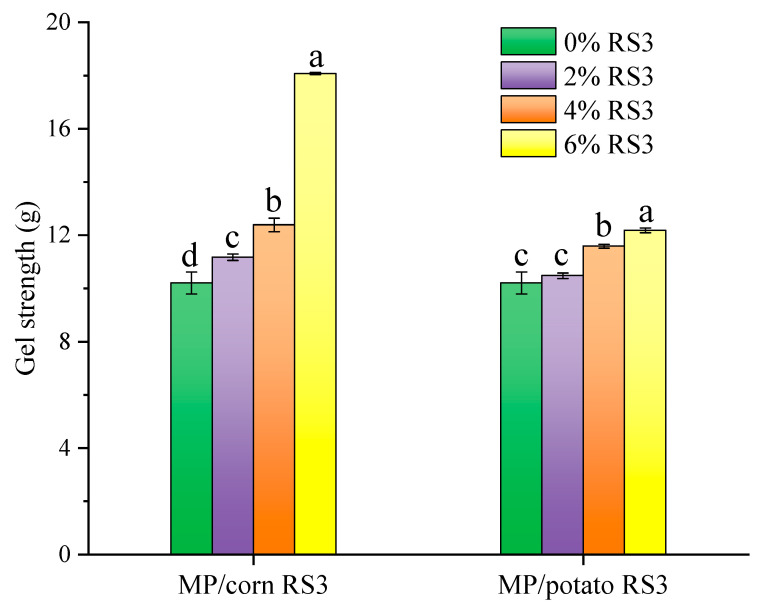
Gel strength of pure MP emulsion gel and MP/RS3 composited emulsion gels. Different letters in each group indicate significant difference (*p* < 0.05).

Gel strength can reflect the ability of proteins to aggregate in an orderly way and form a tight gel network structure upon heating. The gel strength of the emulsion gels stabilized by MP/RS3 mixtures is shown in Figure 9. The gel strength of the composite gels increased by raising the amount of RS3 added, and the gels added with 2–6% RS3 had a significantly higher gel strength than the pure MP gel (*p* < 0.05). In particular, the gel strength of the emulsion gel added with 6% corn RS3 increased by 77% as compared to the pure gel. This was consistent with the results of rheological behavior. The enhancement in gel strength might be due to the following two factors: (1) RS3 absorbed water and swelled under heat induction, and the expanded resistant starch particles could serve as a filler and exert pressure on the MP emulsion gel matrix [4,28]; and (2) the cross-linked aggregation of proteins induced by RS3 addition contributed to the formation of a stronger and tighter gel network structure [21].

**Figure 10:**
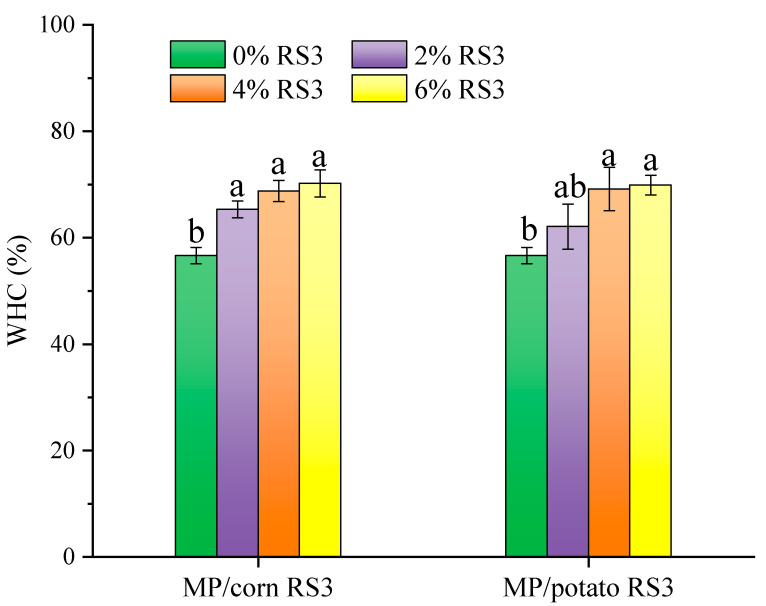
Water holding capacity (WHC) of pure MP emulsion gel and MP/RS3 composited emulsion gels. Different letters in each group indicate significant difference (*p* < 0.05).

WHC is a key indicator for reflecting the ability of protein gel to hold water, and ultimately affects the edible quality and economic value of meat and meat products. The WHC values of the emulsion gels stabilized by MP/RS3 mixtures were determined and are shown in Figure 10. Similar to the results of gel strength, the emulsion gels added with RS3 had a remarkably higher WHC value than the pure MP gel (*p* < 0.05). The increased WHC indicated that RS3 reinforced the ability of the MP emulsion gel to retain water. A previous study also found that cassava starch was capable of strengthening the gel network of silver carp surimi, embedding more water in the gel matrix [29]. In addition, the hydrogen bonding between highly hydrophilic RS3 and water molecules might account for the enhanced WHC [4]. Moreover, based on previous studies, the indigestible portion of carbohydrates could help to improve the WHC of MP gels [30,31], which further confirmed our findings.

#### 3.5.4. Texture Profile Analysis

**Figure 11:**
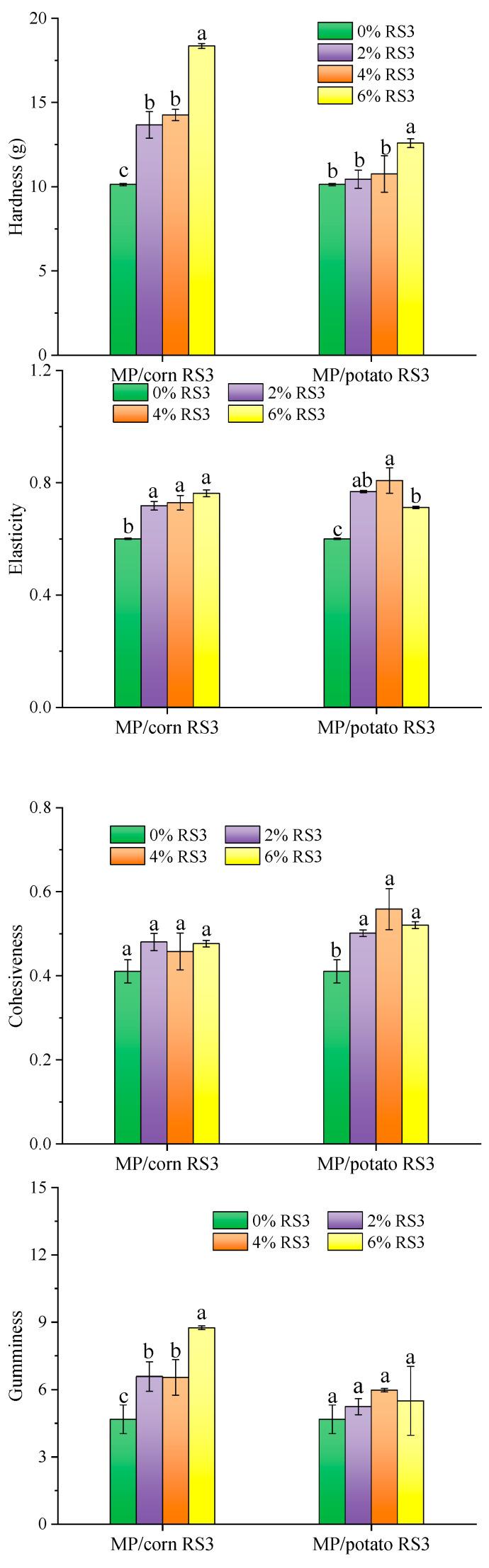

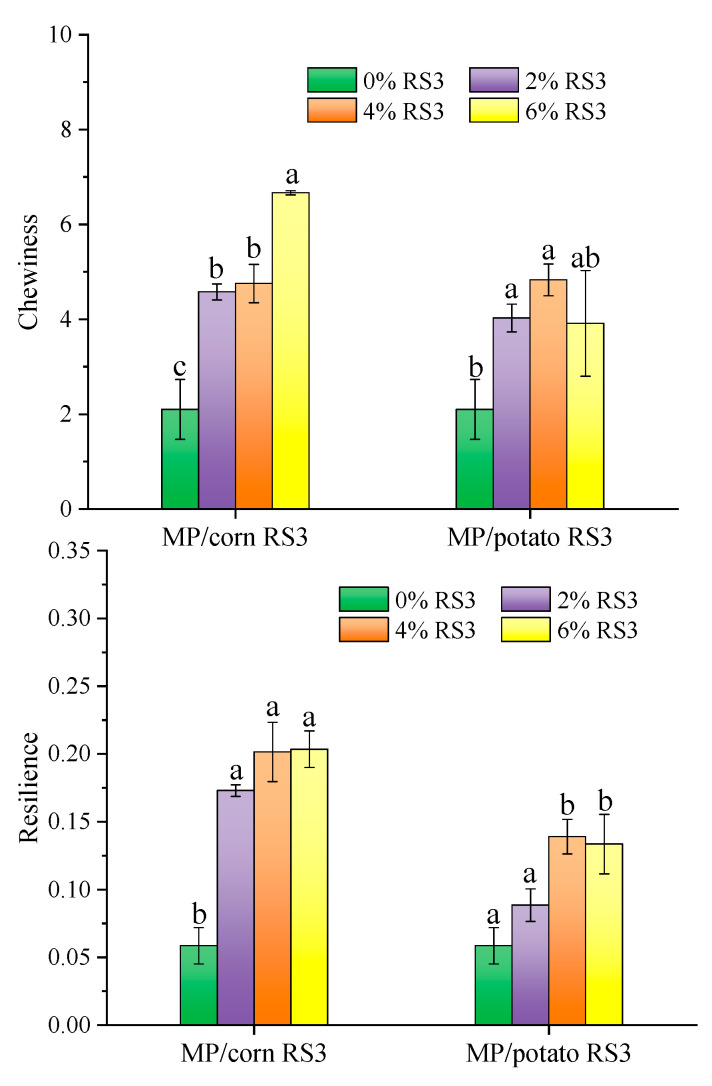
Textural properties of pure MP emulsion gel and MP/RS3 composited emulsion gels. Different letters in each group indicate significant difference (*p* < 0.05).

Texture is an important property for meat processing, which is mainly affected by the source and composition of meat and the interactions between additives and meat proteins during the formation of heat-induced gel [30]. The textural properties of the emulsion gels stabilized by MP/RS3 mixtures are shown in Figure 11. The hardness, elasticity, cohesiveness, gumminess, chewiness and resilience of the emulsion gel were all enhanced by RS3 addition. The gel added with 2% corn RS3 had a significantly higher hardness value than the pure gel, while the hardness of the gel added with 2–4% potato RS3 was comparable to that of the pure gel (*p* > 0.05), indicating that the improvement effect of corn RS3 on gel structure was stronger than that of potato RS3, which was in accordance with the G′ (Figure 8) and gel strength (Figure 9) results. This might be attributed to the higher amylopectin content of potato RS3. Pietrasik and Soladoye [32] showed that bologna added with pea starch alternative to modified corn starch (MCS) had higher hardness and chewiness since MCS rich in amylopectin tended to form soft gels. A previous study indicated that unripe banana flour enhanced the hardness of MP gel mainly because of its resistant starch and fiber [30]. The expanded RS3 particles may absorb water that was released from protein denaturation during heating, and fill the MP emulsion gel to form a more compact and firmer structure [32], which reduced the water mobility and the voids within the gel matrix and thus improved the hardness, springiness and other textural performances.

#### 3.5.5. Moisture Distribution

**Figure 12:**
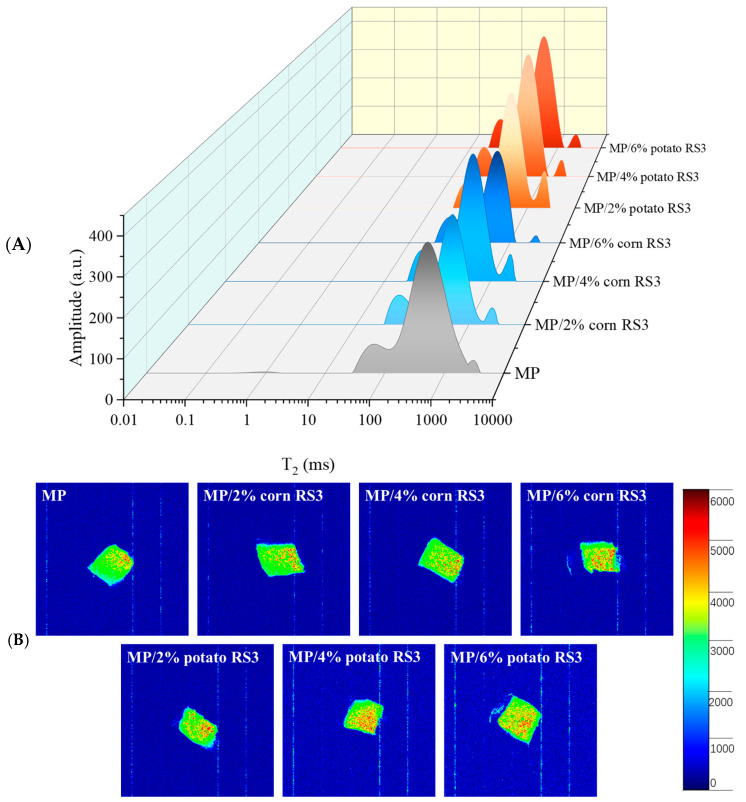
*T*_2_ relaxation time distribution curves (**A**) and MRI images (**B**) of pure MP emulsion gel and MP/RS3 composited emulsion gels.

LF-NMR is a powerful tool to determinate the water state in gel through monitoring T_2_ relaxation time. Figure 12A shows the distribution curves of the T_2_ relaxation time of the emulsion gels stabilized by MP/RS3 mixtures. Three water populations were detected, located at about 20–100 ms (T_21_), 100-1000 ms (T_22_) and 1000–4000 ms (T_23_), respectively, corresponding to bound water, immobilized water and free water [28]. After adding RS3, the position of T_2_ shifted toward a shorter relaxation time, indicating that more water was associated with the MP emulsion gel. In particular, the T_22_ values obviously decreased to 1647.32~2410.86 ms (for corn RS3) and to 1536.83~1686.73 ms (for potato RS3), respectively, which suggested that RS3 addition facilitated the migration of free water to immobilized water and further improved the water retention of the MP gels. This could be attributed to the strengthening of the gel network’s ability to hold water and the excellent water absorption ability of RS3. These findings were consistent with the enhanced WHC (Figure 10). The water distribution in the emulsion gels was intuitively observed by MRI. In general, the higher the brightness of yellow and red pixels, the higher the density of water protons in the gel and the more immobilized water [15]. As shown in Figure 12B, by increasing the amount of RS3 addition, more water molecules were trapped in the gel matrix, exhibiting a larger bright area in the image, which corresponded to the changes in WHC and T_22_.

#### 3.5.6. Molecular Forces in Gels

**Figure 13:**
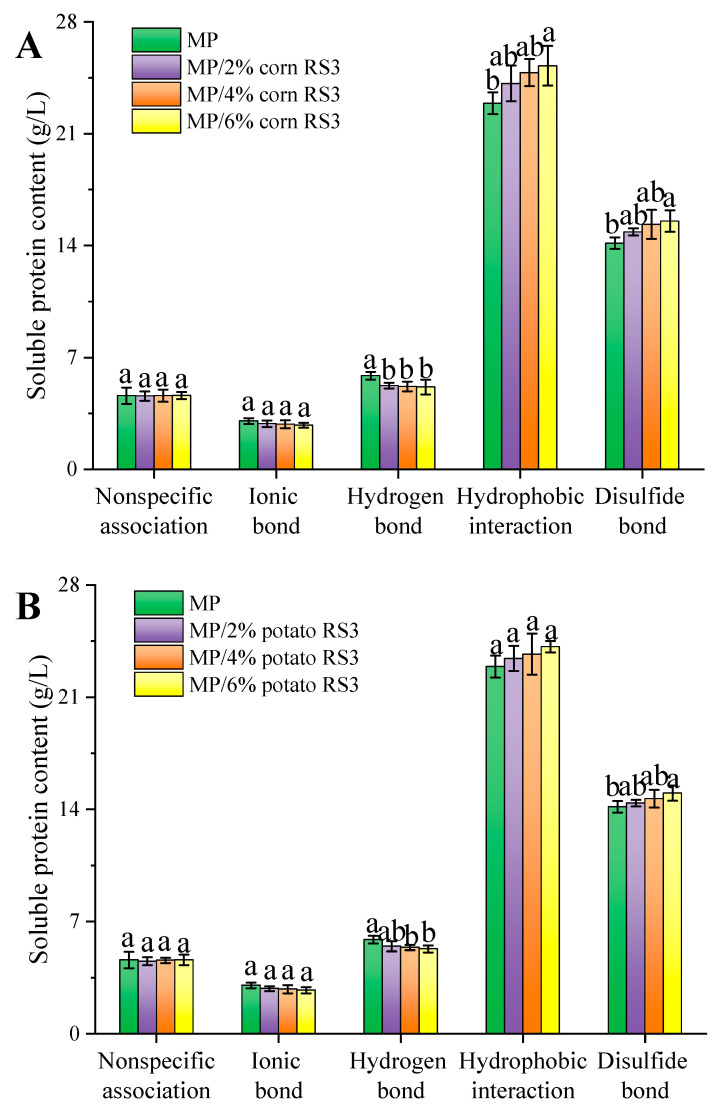
Molecular forces of pure MP emulsion gel and MP/RS3 composited emulsion gels. (**A**): corn RS3, and (**B**): potato RS3. Different letters in each group indicate significant difference (*p* < 0.05).

The equilibrium of molecular forces including hydrophobic interaction, hydrogen bonding, ionic interaction and disulfide linkages played an important part in protein gelation. Protein solubility was used to evaluate these forces by dissolving the MP/RS3 composite emulsion gels in different chemicals. As shown in Figure 13, hydrophobic interaction and disulfide bonds were the main forces stabilizing the gel structure. The hydrophobic interaction and disulfide bonds formed via the exposed hydrophobic region and active sulfhydryl groups promoted the aggregation of heat-denatured proteins and contributed to the formation of a stable gel network structure [2]. As the amount of RS3 added increased, the hydrophobic interaction and disulfide bonds were strengthened, while the hydrogen bonding decreased, demonstrating that RS3 might alter the spatial conformation of the proteins by affecting their interactions. The decrease in the level of hydrogen bonding was probably due to the higher affinity between water and RS3, which was rich in hydrophilic groups, thus weakening the binding of water to MP molecules.

#### 3.5.7. Microstructure

The structure of emulsion gel is closely related to the distribution of internal oil droplets [17]. The oil distribution in the MP gel networks was characterized using confocal laser scanning microscopy (CLSM). Based on the above characterization, the MP emulsion gels added with 2% and 6% RS3 were selected for CLSM observation in order to compare their microstructure differences more obviously. As shown in Figure S1, MPs stained with Nile blue and the oil droplets stained with Nile red exhibited blue and green fluorescence, respectively. The pure MP emulsion gel showed a slightly aggregated droplet structure with irregular sizes. The addition of 2% RS3 obviously reduced the quantity of large oil droplets, indicating an improved gel network. As the amount of RS3 added increased to 6%, a more uniform distribution of the emulsion droplets with smaller sizes was observed. These findings could be explained in that a homogeneous and well-formed gel network was formed due to enhanced interactions between the proteins in the continuous phase (non-adsorbed) and the interfacial proteins (adsorbed), which limited the movement and flocculation/coalescence of the oil droplets and allowed the droplets to be regularly embedded in the protein gel network. This was confirmed by the results of surface hydrophobicity (Figure 2) and intrinsic tryptophan fluorescence (Figure 3). These changes were consistent with the increase in gel strength (Figure 9) and WHC (Figure 10). A previous study also found that the addition of starch facilitated the formation of a better MP gel network with oil droplets more evenly dispersed in the protein matrix, thus improving gel strength and texture [18].

#### 3.5.8. General Discussion

**Figure 14:**
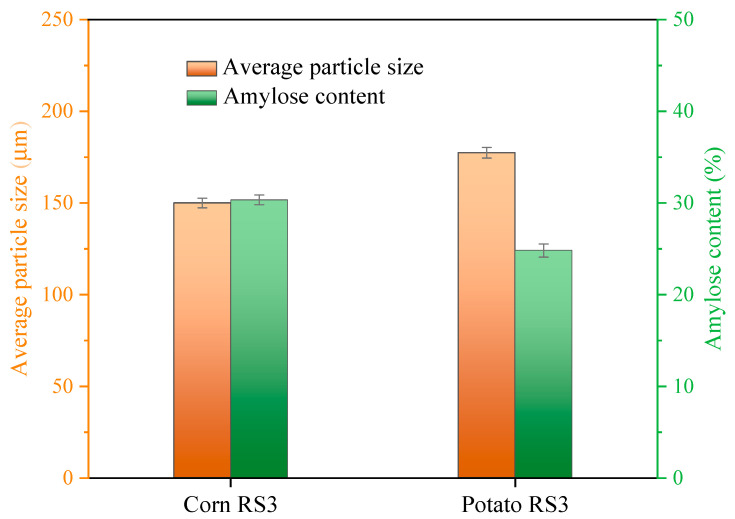
Average particle size and amylose content of corn RS3 and potato RS3.

Overall, the MP emulsion gel added with corn RS3 had much higher G’ and gel strength and better textural properties than that added with potato RS3. This could be related to the physicochemical properties of the starches. Figure 14 shows the average particle size and amylose content of corn RS3 and potato RS3. Corn RS3 had a smaller average size and a higher amylose content than potato RS3. Generally, smaller-sized starch particles may be more beneficial as a way to improve the gel properties of MPs since starch granules with a smaller particle size can be more uniformly dispersed in the MP gel matrix to form a stronger network structure, leading to enhanced two-phase compatibility with higher G′ and gel strength [33]. Conversely, large starch particles may interfere with the continuity of the MP network and reduce the associations between the proteins. Similar results were obtained when nano-sized and micro-sized okara dietary fibers were added to silver carp surimi gels separately [34]. Therefore, compared with potato RS3, corn RS3 with a relatively smaller particle size may be more readily incorporated into the MP gel network and interpenetrate with the proteins, thus effectively strengthening the gel texture. In addition, previous studies have demonstrated that the amylose moiety of starch is responsible for the gel strength [32], and a higher portion of amylose contributed to the reinforcement of gel texture [35]. This could be due to the difference in the molecular structures of amylose and amylopectin. Amylose has a moderate molecular weight, with a relatively spiral linear molecular structure and a small number of long-chain branches, while amylopectin possesses a multi-branched structure with a great number of short branches and relatively large molecular weight [36]. These factors may affect the texture of protein-based gels. To be specific, corn RS3 rich in amylose may favor the entanglement with the MP network, which facilitated the establishment of an intercohesive bonding between the starch granules and MPs to form a supramolecular gel structure with higher gel strength and WHC [35]. The distinct molecular structure of amylose and amylopectin usually imparts foods with different textures. Foods with higher amounts of amylose always have a harder texture [37], while those containing a greater amount of amylopectin tended to form soft gels [32]. In this paper, corn RS3 rich in amylose exhibited an excellent texture-improving ability, and it was thus not difficult to understand why the improvement effect of corn RS3 on the gel quality of MP was superior to that of potato RS3 with a relatively low amylose content.

### 3.6. In Vitro Digestion of Emulsion Gels

#### 3.6.1. Digestibility

**Figure 15:**
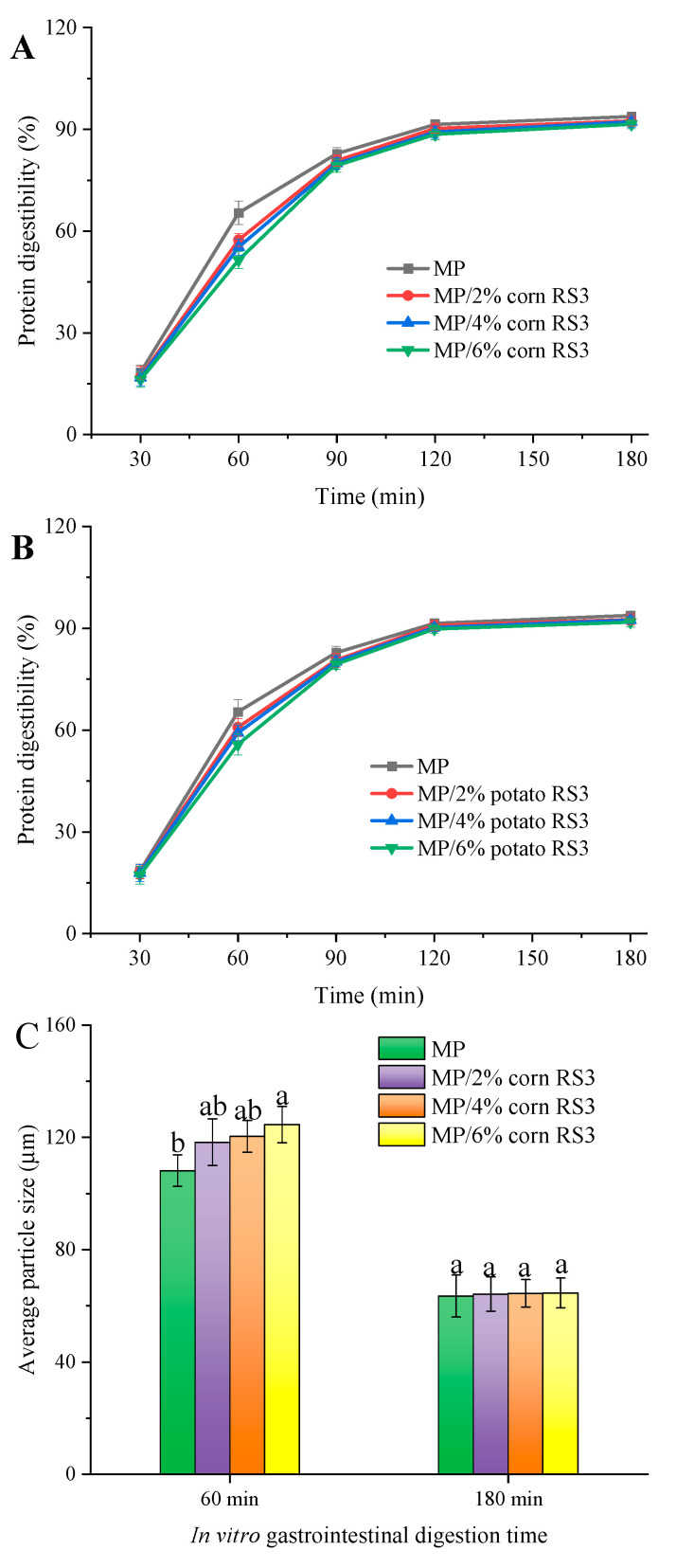

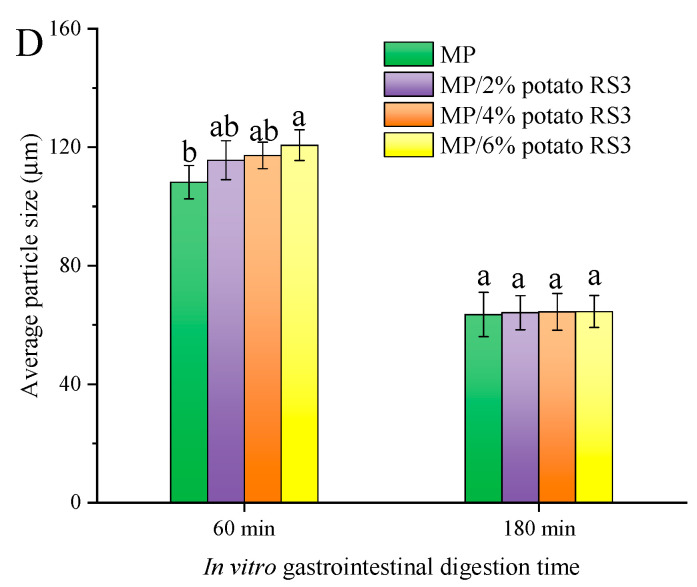
The in vitro digestibility (**A**,**B**) of MP emulsion gels at each time point (30, 60, 90, 120, 180 min), and the average particle size (**C**,**D**) of digests after saliva/gastric digestion and saliva/gastric/intestinal digestion, respectively. Different letters in each group indicate significant difference (*p* < 0.05).

Figure 15A,B show the changes in the in vitro protein digestibility of composite emulsion gels from 0 to 182 min (2 min for saliva digestion +60 min for gastric digestion + 120 min for intestinal digestion). During the saliva/gastric digestion, the protein digestibility of the gels treated with RS3 was lower than that of the pure gel, especially at higher RS3 addition levels. For instance, the protein digestibility after the saliva/gastric digestion significantly decreased to 51.5~55.8% for the gels composited with 6% RS3 (*p* < 0.05). This might be related to the physical barrier effect of the starch granules [38]. The added RS3 filled the protein network and blocked the cleavage sites available for digestive enzymes, thereby delaying the digestion of protein. In addition, gel hardness was reported to be negatively correlated with the gastric digestion of protein gel [39]. This explained why the hard gels composited with 6% RS3 (as indicated in Figure 11) had a decreased proteolytic sensitivity and a lower pepsin digestibility. After intestinal digestion, the protein digestibility of all gel samples was >90%, indicating that the presence of RS3 did not change the overall digestibility of MPs. Generally, a dramatic decrease in digestibility is linked to excessive aggregation of proteins or a significant loss of enzyme-targeted amino acid residues [40], which are not present in this paper. Furthermore, the greater digestibility in the simulated intestinal phase could be explained by trypsin, as a nonspecific endopeptidase, having a higher hydrolysis efficiency for breaking down protein aggregates than pepsin. These findings suggest that it is possible to design functional muscle gel-based foods with satiation by adding RS3 to delay the gastric digestion of muscle proteins without influencing their intestinal digestibility.

#### 3.6.2. Particle Size

Particle size indicates the degradation degree of meat proteins during gastrointestinal digestion. After saliva/gastric digestion, the average particle size of all gel samples decreased (as indicated in Figure 15C,D). However, the reduction in the average particle size of the gels added with high levels of RS3 was lower than that of the pure MP gel. As expected, the pepsin/trypsin treatment led to a further decrease in average diameter, and there was no significant difference (*p* > 0.05) in the final particle size of all protein gel samples. These results indicated that RS3 addition had a negligible impact on the degradation of proteins into small particles by pepsin/trypsin, which was in accordance with the changes in in vitro protein digestibility. It was also inferred that resistant starch may reduce the production of heat-induced oxidation products by improving the antioxidant capacity of muscle gel, which helped to obtain small-sized protein digests [38].

### 3.7. Statistical Correlation Analysis

**Figure 16:**
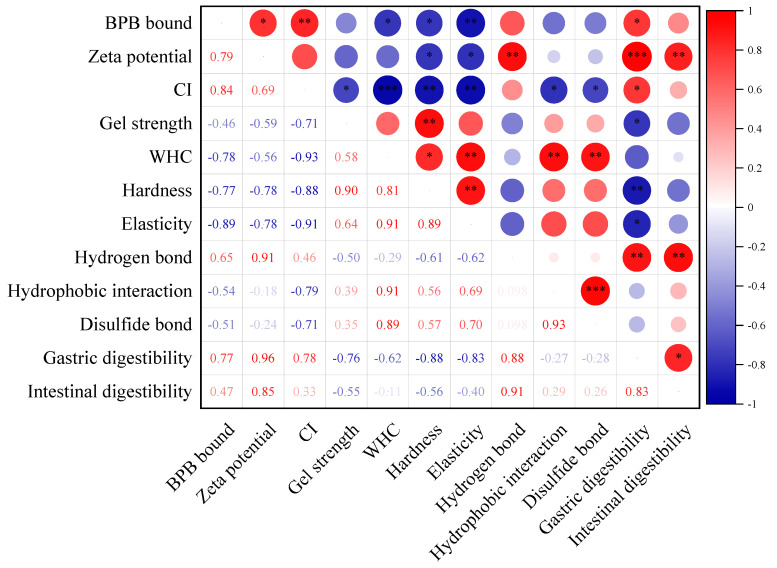
Correlation study among MP emulsion stability, emulsion gel properties and in vitro protein digestibility. * *p* < 0.05, ** *p* < 0.01, and *** *p* < 0.001.

Figure 16 shows the correlation analyses between the MP emulsion stability, emulsion gel properties and in vitro protein digestibility. A high negative correlation was observed (*p* < 0.01) between CI and emulsion gel properties (WHC, hardness and elasticity), indicating that the properties of MP emulsion gel were closely related to the stability of the emulsion before heating. In addition, hydrophobic interaction and disulfide bonding were positively correlated with WHC. This implied that the MP gel network, which was mainly maintained by hydrophobic force and disulfide linkage during heating, could retain a large amount of water and thus improve the WHC of the gel. It was also found that the gastric protein digestibility was highly negatively correlated with the hardness of the emulsion gel (*p* < 0.01), which further confirmed that the presence of RS3 delayed the gastric digestion of the proteins in gel. Overall, RS3 addition improved the stability of the MP-based emulsion, which facilitated the formation of a well-organized gel network with oil droplets better anchored and thus enhanced the gel performance of MP. Moreover, the presence of RS3 did not affect the final intestinal digestion of MP.

## 4. Conclusions

This study demonstrated that the emulsion stability and emulsion gel properties stabilized by MPs were improved by the addition of corn RS3 or potato RS3. The presence of RS3 promoted protein–protein interactions, as evidenced by the decreased surface hydrophobicity (*p* < 0.05) and increased fluorescence intensity of MPs. Thus, a stable cross-linked protein network might be formed, which limited the migration of oil droplets and improved the stability of the emulsion. Upon heating, RS3 filled in the interstitial spaces of the MP network, and bound water and fat to form a stronger gel structure with higher elasticity, gel strength and WHC, more immobilized water and improved textural properties. Meanwhile, the hydrophobic interaction and disulfide bonds between MPs were enhanced, especially at 6% RS3 added. Furthermore, the addition of RS3 resulted in a relatively lower pepsin digestibility but did not influence the overall protein digestibility of the gels. Future work will focus on the in vivo digestion behavior of the composite gels and the application of RS3 in meat products. Our findings indicate that the incorporation of RS3 is a promising method to produce high-quality low-GI meat products without damaging the nutritional value of protein.

## Data Availability

The original contributions presented in the study are included in the article/Supplementary Materials, further inquiries can be directed to the corresponding author.

## Supplementary Materials

The following supporting information can be downloaded at: https://www.mdpi.com/article/10.3390/foods13233739/s1, Figure S1. CLSM images of pure MP emulsion gel and MP/RS3 composited emulsion gels.
