# Supplementary material for: Retrograded Resistant Starch Improves Emulsion Stability and Emulsion Gel Properties Stabilized by Myofibrillar Proteins Without Degrading In Vitro Protein Digestibility
Source: Foods. 2024 Nov 22;13(23):3739. doi: 10.3390/foods13233739 (PMC11640666; doi:10.3390/foods13233739)
Supplement: Supplementary file 1 [file foods-13-03739-s001.zip › foods-3299725-supplementary.pdf]

Supplementary Data

Retrograded resistant starch improves emulsion stability and emulsion gel properties stabilized by myofibrillar proteins without degrading *in vitro* protein digestibility

Jinyu Chen[a,b,*], Fangyang Hu[a,c], Jiaqi Guo[a], Wen Zhang[a,b], Zijian Wu[a,b]

[a]College of Biotechnology and Food Science, Tianjin University of Commerce, Tianjin 300134, China
[b]Tianjin Key Laboratory of Food Biotechnology, Tianjin 300134, China
[c]Agriculture and Food Engineering College, Baise University, Baise 533000, Guangxi, China

*Correspondence: Jinyu Chen (E-mail: 920162744@tjcu.edu.cn)

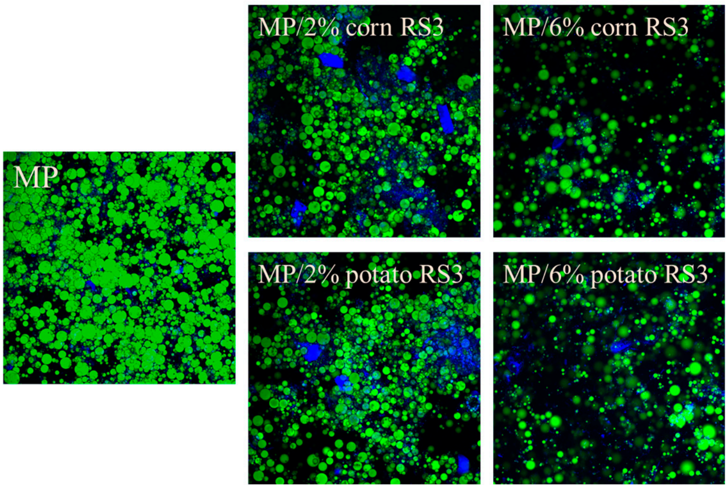

Figure S1. CLSM images of pure MP emulsion gel and MP/RS3 composited emulsion gels.
